# Synergistic Effects of Bacteriocin from *Lactobacillus panis* C-M2 Combined with Dielectric Barrier Discharged Non-Thermal Plasma (DBD-NTP) on *Morganella* sp. in Aquatic Foods

**DOI:** 10.3390/antibiotics9090593

**Published:** 2020-09-10

**Authors:** Chengjun Shan, Han Wu, Jianzhong Zhou, Wenjing Yan, Jianhao Zhang, Xiaoli Liu

**Affiliations:** 1 College of Food Science and Technology, Nanjing Agricultural University, Nanjing 210095, China; 20060004@jaas.ac.cn (C.S.); ywj1103@njau.edu.cn (W.Y.); 2 Institute of Agro-Product Processing, Jiangsu Academy of Agricultural Sciences, Nanjing 210014, China; wuhan@jaas.ac.cn (H.W.); 20060005@jaas.ac.cn (J.Z.)

**Keywords:** bacteriocin, non-thermal plasma, molecular mechanism, putrefactive bacteria, aquatic foods

## Abstract

In this paper, Lactocin C-M2(C-M2) was used together with a new non-thermal technology, non-thermal plasma sterilization (NTPS), to inactive the putrefactive bacteria *Morganella* sp. wf-1 isolated from aquatic foods. The mechanism underlining the action mode of C-M2 and NTPS was investigated, revealing that the bacteriocin and NTPS had synergistic effects on the disinfection of *Morganella* sp. wf-1. Compared with the bacteria cells treated by only C-M2 or NTPS, the plasmolysis of cells treated by C-M2 and NTPS was to a larger extent. Moreover, the cell permeability and the contents of UV-absorbing compounds and K^+^ released from the intra-cells was significantly higher for the C-M2 + NTPS treated cells than the others (*p* < 0.05), and conversely was the SFA/UFA ratio (*p* < 0.05). The results on DNA damage showed that, 8-hydroxy-2′-deoxyguanosine(8-OHdG) content in C-M2 + NTPS treated cells was approximately 7 -fold and 2.5-fold greater than those in the C-M2- and NTPS-treated cells, respectively, indicating furthermore the eventual rupture of *Morganella* sp. wf-1 cells. The results showed the potential of the application of the bacteriocin and NTPS in the food industry.

## 1. Introduction

Nowadays, a health hazard to consumers arises due to the possible presence of microbial toxins as a consequence of food contamination with spoilage bacteria [1]. *Morganella* species are Gram-negative, rod-shaped, aerobic, and facultatively anaerobic, which can be isolated from frozen and non-frozen aquatic products, such as vacuum-packed or cold-smoked tuna, or mackerel stored at 30 °C [2,3]. Some strains have been identified as histamine producing bacteria, and implicated in incidents of histamine fish poisoning [4]. Although *Morganella* has low pathogenicity, some species act as opportunistic pathogens, resulting in urinary tract infections in humans through the adhesion and secretion systems [5].

In view of different methods used for the inactivation of the putrefactive bacteria in foods, there exist some disadvantages. Only less than 2 log_10_ unit reductions of pathogens may be caused by the postharvest washing and sanitising treatments [6]. Moreover, based on heating process, the conventional thermal methods facilitate a mass transfer between different phases of system and consume lots of energy, significantly changing the concentration, bioavailability, and bioactivity of phytochemicals in food matrix [7,8]. As for some low pH-based preservation techniques, they may contribute to the bacterial adaption to an acidic environment and subsequently increase their acid resistance. Thus, the situation has initiated a search for more effective and greener biopreservatives or techniques.

Bacteriocins are ribosomally synthesized proteins against related bacteria or across genera. They generally show low eukaryotic toxicity and can be used in food industry as natural preservatives. Their proteinaceous nature implies the putative degradation in the gastro-intestinal tract of animals. Particularly, some bacteriocins have a remarkable therapeutic potential in both local and systemic bacterial infection. Lactic acid bacteria (LAB)-produced bacteriocins have attracted a rising attention because they can be used as natural food biopreservatives for improving the food safety [9].

There exist numerous studies on the mode of action of LAB-produced bacteriocins against Gram-positive bacteria [10]. To date, there still exist a few bacteriocins showing broad-spectrum activity that are available to use, including the plantaricin ST31, bacteriocin AMA-K, plantaricin MG, sakacin C2, ent35-MccV, lactocin MXJ 32A, and bifidocin A [11]. An understanding about the effects of these bacteriocins against Gram-negative bacteria is necessary for their application [12].

Lactocin C-M2, a novel 863.52 Da bacteriocin, was produced by *Lactobacillus panis* C-M2, which was conserved in the Food Bioengineering Laboratory, Jiangsu Academy of Agricultural Sciences, China. In our previous published work [13], the Lactocin was purified by SP-Sepharose Fast Flow, SDS–PAGE, and HPLC, and its N-terminal region sequence was identified as Met-Val-Lys-Lys-Thr-Ser-Ala-Val. This bacteriocin showed an obvious inhibitory activity against various bacteria. After sterilization at 121 °C for 15 min, the residual activity of C-M2 maintained at a level of 82.1%, and only 0.2% and 14.4% reduced at pH 2–3 and pH 6, respectively. Its heat-stable and pH-resistant characteristics, along with the broad spectrum of antibacterial activity, suggested great potential as a biopreservative in the food industry.

Meanwhile, the non-thermal processing has the ability to inactivate microorganisms at ambient temperatures to avoid the negative effects of heat on flavor, color, and nutritional value of foods, consequently making it a promising technique to meet consumer’s requirements [14]. Among them, the non-thermal plasma is a relatively innovative non-thermal process, and the food industry is beginning to recognize its potential as a sterilization method [6]. As the fourth state of matter, the plasma is a partially or completely ionized gas and a reactive atmosphere where a variety of energetic and charged species, radicals, neutral species, and photons are formed mainly from the collision of energetic electrons with heavy particles, breaking down the covalent bonds and initiating numerous chemical reactions [15]. The reactive oxygen species are known to be the key components to provide plasma inactivation, cell damage, and cell death [16]. They are responsible for the biological reactions ranging from intercellular DNA fracture and protein degeneration to oxidation of the outer membrane [17]. Moreover, the charged particles accumulate on the surface of the cell membrane and induce the subsequent rupture.

The non-thermal plasma operation is accompanied by the low temperature and simultaneous high antimicrobial effects, which leads it to be regarded as a future alternative for thermal pasteurization [18]. According to some other reports, the plasma technology showed promise as a direct treatment for fresh and fresh-cut fruits and vegetables, as well as for nuts and other foods [19]. The plasma effectively killed the bacteria on food surfaces but did not significantly affect the quality of treated products. The antimicrobial efficacy and design of ACP systems, including producer gas composition, electrode configuration, as well as the type of bacteria and substrate, varies widely [20]. However, the biggest challenge about the application of non-thermal technologies for food processing is the inactivation of pathogenic microorganisms and spoilage agents, which can be overcome by using various methods. However, there are few studies investigating the possibility of using the non-thermal plasma combined with antimicrobial solution for the inactivation of bacterial pathogens [21]. It is still necessary to understand the internal mechanism of bacterial disinfection caused by the non-thermal plasma treatment synergistically with the bacteriocin.

In the present study, the action mechanism of Lactocin C-M2 and non-thermal plasma was studied on *Morganella* sp. strain wf-1 which is isolated from white fish in our previous study. *Morganella* sp. is one of the most susceptible putrefactive bacteria in aquatic food. The primary objective of this study was to investigate the use of antimicrobials and non-thermal plasma combination treatments to reduce the inoculated *Morganella* sp. bacteria, with particular attention directed to the bacterial populations. Then, the morphology, cell membrane permeability, integrity (e.g., leakage of UV absorbing materials), and DNA damage of the bacteria cells were furthermore determined.

## 2. Results

### 2.1. Effects of C-M2 and NTPS on the Growth Inhibition of Morganella sp. wf-1

#### 2.1.1. Treatments with Different Concentrations of C-M2

**Figure 1:**
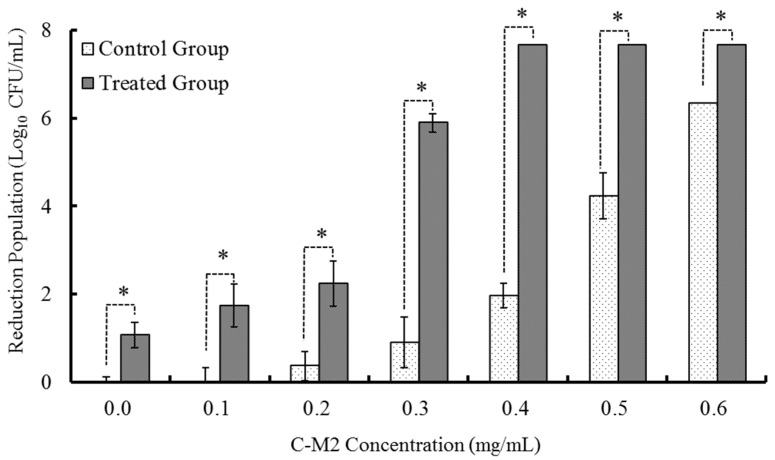
Inhibitory effect of bacteriocin Lactocin C-M2 on *Morganella* sp.wf-1. Statistically significant finding at * *p* < 0.05 compared with their respective controls of same concentration.

As shown in Figure 1, different concentrations (0, 0.1, 0.2, 0.3, 0.4, 0.5, 0.6 mg/mL) of Lactocin C-M2 without and along with the fixed non-thermal plasma treatment (65 kV, 90 s) were used to sterilize *Morganella* sp. wf-1 in the control and experimental groups, respectively. There was a positive correlation between the C-M2 concentration and the mortality of bacteria for both the two groups (*R*^2^ = 0.8465 and 0.8812 for the control and experimental, respectively). Moreover, the NTPS treatment could significantly enhance the effects of C-M2 on the sterilization of *Morganella* sp. wf-1 (*p* < 0.05). When the additive ratio of C-M2 was of 0.3 mg/mL, the amount of bacteria sterilized was 5.76-fold in the experimental group compared with that in the control. The C-M2 concentration of 0.3 mg/mL was the turning point for sterilizing the bacteria in the treatment without and with NTPS. When the C-M2 concentration was more than 0.4 mg/mL, there was no significant increase in the mortality in the experimental group. However, the mortality in the control group increased rapidly.

#### 2.1.2. Treatments with Different times of CSP

As above, *Morganella* sp. wf-1 were divided into two groups, namely the control group and the experimental group, which were treated by the NTPS with different durations (0, 30, 60, 90, 120, 150, 180 s) combined without and with the addition of Lactocin C-M2, respectively. The number of dead bacteria significantly increased as the duration of NTPS augmented (*p* < 0.05). Compared with the bacteria treated by NTPS only, bacteria samples treated by the NTPS following the addition of C-M2 had the significantly higher rates of mortality (*p* < 0.05).

**Figure 2:**
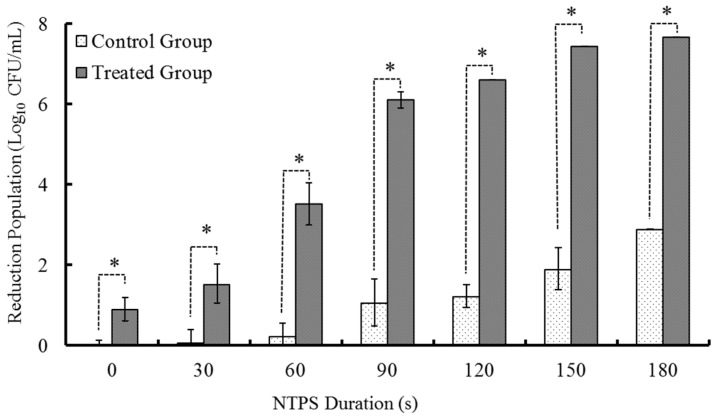
Inhibitory effect of NTPS processing time on spoilage bacteria. Statistically significant finding at * *p* < 0.05 compared with their respective controls of same duration.

As indicated in Figure 2, when the NTPS treatment time increased from 90 s to 120 s, the mortality of *Morganella* sp. wf-1 was aggrandized by approximately 4.5 times under the condition of synergistic treatment of C-M2 and NTPS. After being treated by NTPS for 150 s and 180 s, the elimination of viable bacteria varied insignificantly (*p* > 0.05) with the treatment of both C-M2 and NTPS, while that in control groups still increased greatly, which is in coincident with the tendency occurred in Figure 1. Therefore, based on the effects of single factors including lactocin concentration and non-thermal plasma duration time on the sterilization of *Morganella* sp. wf-1, the synergic treatment of C-M2 and NTPS, respectively with 0.3 mg/mL and 120 s were considered to perform for the further experiments.

### 2.2. Effects of Lactocin C-M2 and NTPS on the Morphology

**Figure 3:**
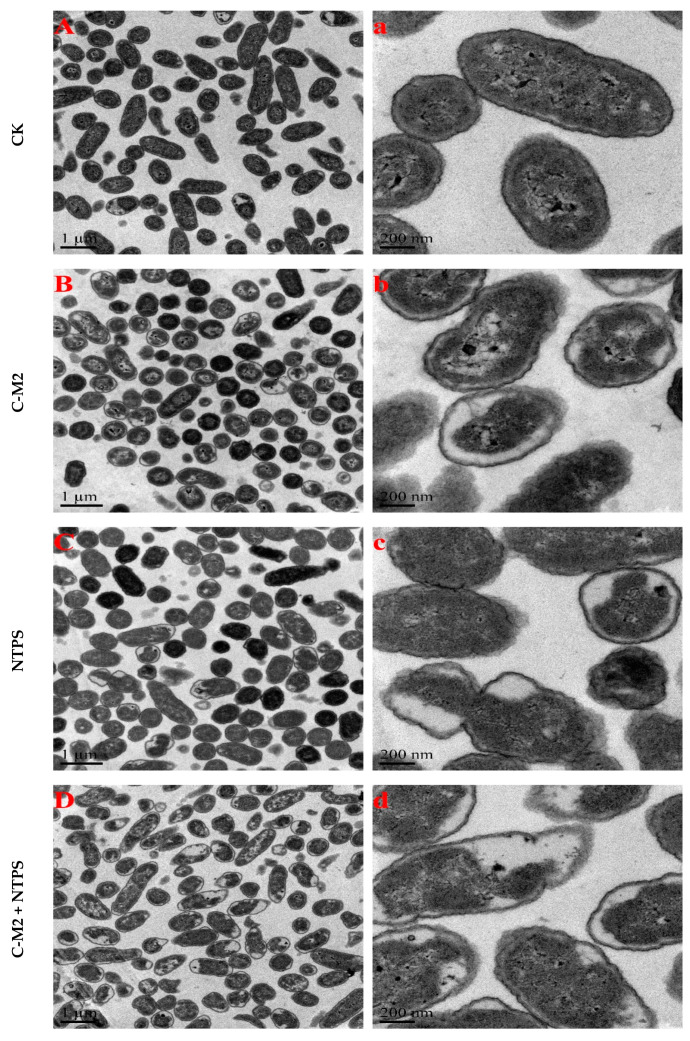
Transmission electron microscopy of *Morganella* sp. wf-1 cells with different treatments. (**A**,**a**) represent untreated control cells; (**B**,**b**), (**C**,**c**) and (**D**,**d**) represent cells treated by C-M2, NTPS and CM2 + NTPS, respectively. Scale bars in (**A**–**D**) = 1 μm; scale bars in (**a**–**d**) = 200 nm.

The transmission electron microscopy was used to demonstrate the effects caused by the lactocin C-M2 and NTPS on the bacteria cell structure. Morphological changes of *Morganella* sp. wf-1 with different treatments were presented in Figure 3. As observed, the untreated control cells of *Morganella* sp. wf-1 showed intact and smooth cell wall and cytoplasmic membrane. The cytoplasm and DNA were evenly distributed in the cell. After exposure to C-M2 and/or NTPS, the disruption of cell membranes occurred. There seemed to be cytoplasm condensation, DNA relaxation, abnormal septation, irregular cross-wall formation, and even cellular lysis. The plasmolysis of *Morganella* sp. wf-1 cells was resulted in a larger extent from the synergistic treatment of C-M2 and NTPS, compared with those in the C-M2 and NTPS groups.

### 2.3. Effects of C-M2 and NTPS on the Cell Membrane Permeability

**Figure 4:**
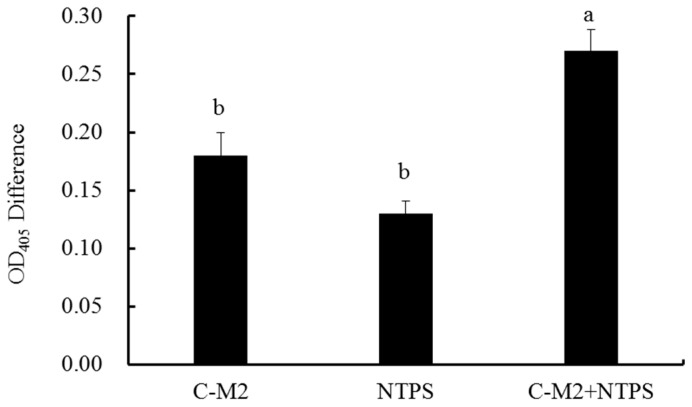
Cell membrane permeability of *Morganella* sp.wf-1 with different treatments. Different lowercase letters (a, b) indicate significant differences (*p* < 0.05).

*Morganella* sp. wf-1 cells were sterilized by three groups of treatment, C-M2, NTPS, and the combination of the two (C-M2 + NTPS). The cell membrane permeability was expressed as the OD_405_ difference between the treated groups and the control. The absolute OD_405_ values, more than zero, indicated the damage on the bacteria cells caused by different treatments (Figure 4), resulting in intracellular enzymes, especially the β-galactosidase, being released into the cytoplasm. Furthermore, the permeability of the group C-M2 + NTPS was significantly higher than the others (*p* < 0.05), which led to the unbalance of the osmotic pressure between the intra- and extra-membrane of bacteria cells and indirectly killed *Morganella* sp. wf-1. Consistent with the findings reported by [22], the investigation on the cell membrane permeability and release of intracellular substances could help to clarify the mechanism of synergistic treatment by C-M2 and NTPS.

### 2.4. Effects of Lactocin C-M2 and NTPS on the Cell Membrane Integrity

#### 2.4.1. Released Cell Nucleic Acid, Protein, and K^+^

**Figure 5:**
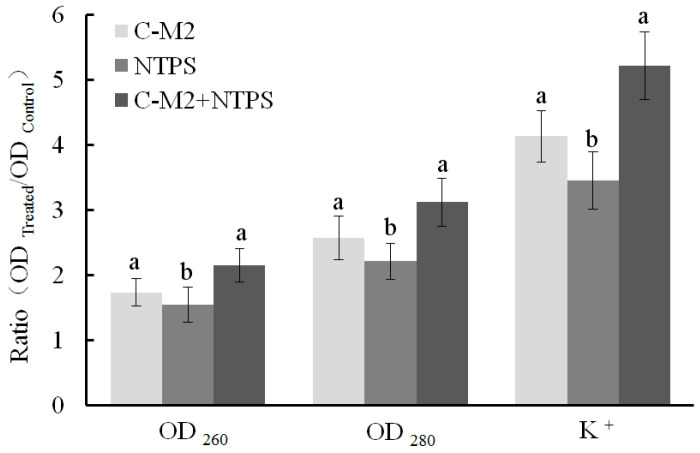
Extracellular UV-absorbing materials and K^+^ released from *Morganella* sp. wf-1 cells with different treatments. Different lowercase letters (a, b) for the same group indicate significant differences (*p* < 0.05).

The cell lysis and non-selective pore formation were reflected by the release of UV-absorbing materials. The significant increases in the extracellular UV-absorbing materials and K^+^ were observed in all the three treated groups (*p* < 0.05, Figure 5). As reported by Suzuki [23], the cells consume available ATP in a futile attempt to re-accumulate inorganic phosphate and K^+^, resulting that the later loss of ATP occurred as a consequence of the early loss of essential ions. Moreover, when the cell membranes were injured after their exposure to different treatments, the K^+^ and phosphates, DNA/RNA, proteins, and enzymes successively entered into the extracellular environment. As shown in Figure 5, the releasing levels of both the nucleic acids (OD_260_) and proteins (OD_280_) were significantly higher for the C-M2 and C-M2 + NTPS groups than the NTPS group (*p* < 0.05), respectively. This result was in accordance with that of the cell membrane permeability for *Morganella* sp. wf-1.

#### 2.4.2. Fatty Acid Composition in Cell Membrane

The fatty acid composition was related with the phospholipid composition and fluidity of bacteria cell membranes. The relative contents of unsaturated fatty acids (UFA) in the cell membranes could increase as the phospholipid was degraded, and was positively correlated with their fluidity. Table 1 presented the composition of saturated and unsaturated fatty acids (SFA and UFA) in cell membranes of *Morganella* sp. wf-1 sterilized in different groups, indicating a significantly higher percentage for the majority of UFA in bacteria in treated groups, compared with that in the control (*p* < 0.05).

**Figure 6:**
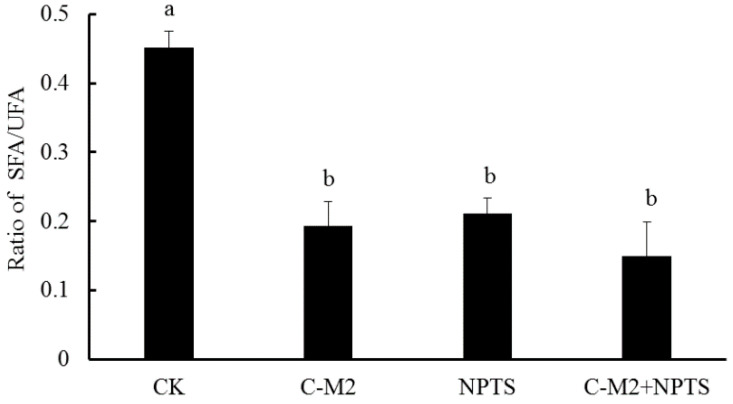
Ratios of SFA/UFA in the cell membrane of *Morganella* sp. wf-1 with different treatments. Different lowercase letters (a, b) indicate significant differences (*p* < 0.05).

Moreover, the ratios of SFA/UFA in the control and treated groups were calculated and shown in Figure 6, demonstrating the highest ration in the control and lower ratios in the treated groups (*p* < 0.05). With respect to the linear chain of SFA and bending chain of UFA, the decrease in SFA/UFA ratio indicated an increase of membrane fluidity. The bacteria cells exhibited the lowest SFA/UFA ratio in the C-M2 +NTPS group, following by the C-M2 and NTPS. Consistently, the cell membranes of *Stenotrophomonas maltophilia* exposed to diesel oil or Triton X-100 also had a decreased SFA/UFA ratio [24]. This current result revealed that the synergistic treatment by lactocin and non-thermal plasma injured the bacteria and had an obvious influence on the cell membrane integrity.

#### 2.4.3. Cell Fluorescent Staining

**Figure 7:**
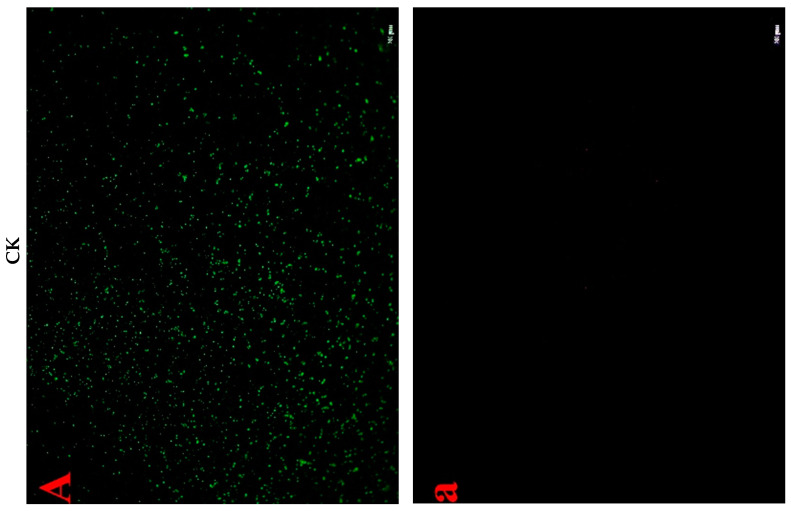

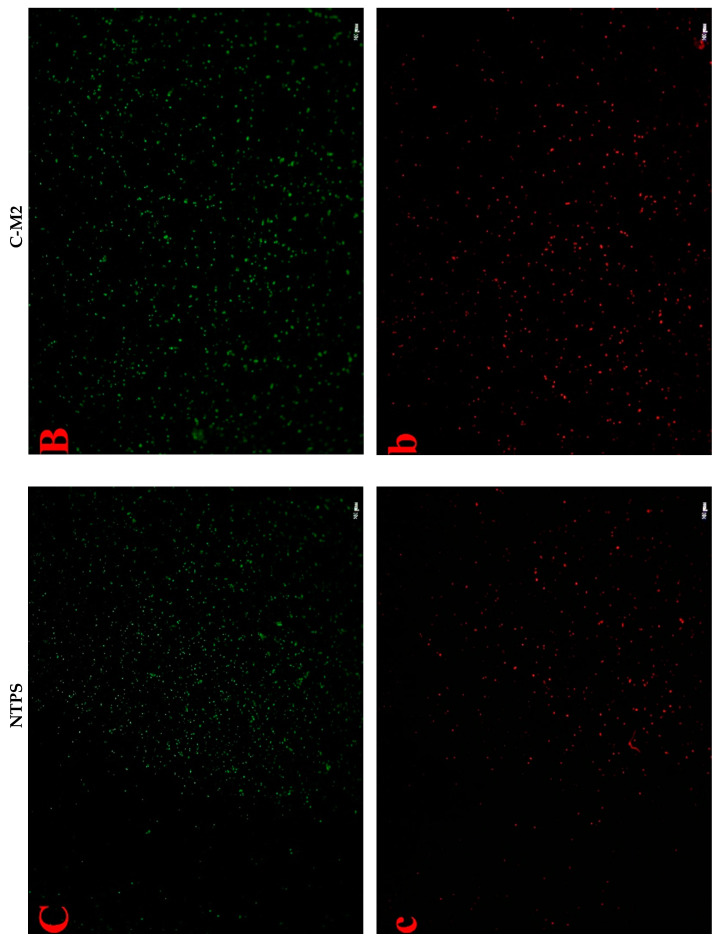

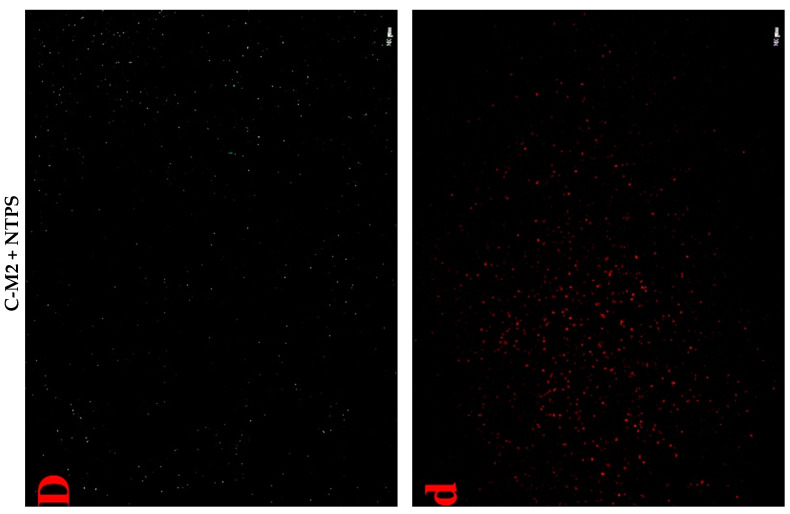
Ratios of SFA/UFA in the cell membrane of *Morganella* sp. wf-1 with different treatments. (**A**,**a**) represent untreated control cells; (**B**,**b**), (**C**,**c**) and (**D**,**d**) represent cells treated by C-M2, NTPS and CM2 + NTPS, respectively. The live cells were stained green and dead cells stained red. Scale bars in figures =20 μm.

The above results were ascertained by LSCM combined with the fluorescent probes, namely SYTO 9 and PI, which distinguish intact cells from membrane-damaged cells. SYTO 9 generally stained all bacteria cells whereas PI penetrates only when the bacteria membrane was damaged. When both dyes were present, a reduction in SYTO 9 fluorescence was caused [25]. Thus, the bacteria cells with intact membranes excluded PI but were stained by SYTO 9 and emitted green fluorescence, whereas those with a damaged membrane were stained by PI and emitted red fluorescence. As shown in Figure 7, the untreated *Morganella* sp. wf-1 cells emitted distinctly more green fluorescence than the treated, and exhibited no red fluorescence. After the synergistic treatment in group C-M2 + NTPS, the red fluorescence intensity of bacteria cells enhanced to be the strongest among all three treated groups, indicating that a large fraction of cells lost their membrane integrity [4].

### 2.5. Effects of Lactocin C-M2 and NTPS on the DNA Damage of Morganella sp. wf-1

**Figure 8:**
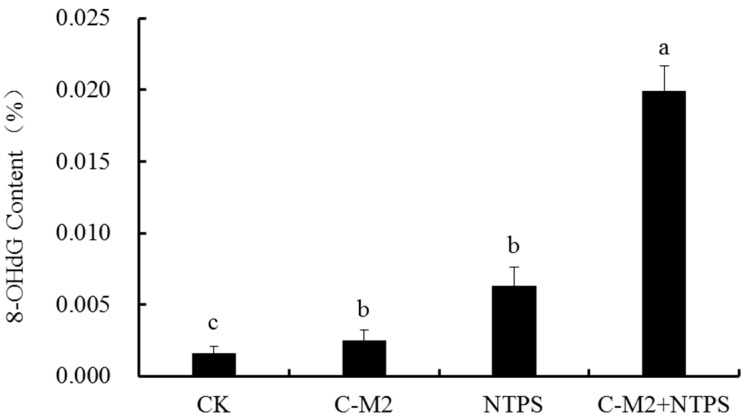
8-OHdG contents produced in *Morganella* sp. wf-1 cells with different treatments. Different lowercase letters indicate significant differences (*p* < 0.05).

DNA damage often occurs as a result of 8-OHdG in the form of ROS-induced (reactive oxygen species, ROS) oxidative stress. Thus, 8-OHdG was widely used as the indicator to determine the DNA damage and mutation in cells. The treatments by the Lactocin C-M2 and NTPS significantly affected the 8-OHdG contents in *Morganella* sp. wf-1 when compared with the untreated group (Figure 8) (*p* < 0.05). In the cells synergistically treated with C-M2 and NTPS, the 8-OHdG content was approximately 7-fold and 2.5-fold more than those of the C-M2 and NTPS groups, respectively. The augmentation of oxidative-stress-related substance indicated that the antioxidant system of *Morganella* sp. wf-1 cells was destroyed, which was probably due to the out-of-balance of the osmotic pressure between intra- and extra-cellular membranes giving rise to more production of ROS, such as hydroxyl radicals and singlet oxygen in cells [26]. Therefore, the combination of lactocin C-M2 and NTPS could contribute more to DNA damage and/or death of *Morganella* sp. wf-1 under oxidative stress than the independent C-M2 or NTPS treatment.

## 3. Discussion

The cell membrane is mainly composed of pore proteins and lipopolysaccharides, playing an important role in separating the intra- and extra-cellular environments, maintaining the basic metabolism of cells, and protecting the cells against external interference. As demonstrated, the bacteriocin of Class I and Class II with a molecular weight less than 10 kDa can bind to the hydrophobic layer or other ions on the surface of cell membrane by electrostatic interaction. The large accumulation of bacteriocin destroys afterwards the membrane in three different ways, namely the pore formation, the micellization, and the fusion of bilayers [27].

In this study, Lactocin C-M2 produced by *Lactobacillus panis* was a small class II peptide containing eight amino acids. The effects of C-M2 on cells could be explained by the theory “pore formation” [28]. Upon the exposure to bacteria, C-M2 quickly bound with the negatively charged sits on the surface of cell membrane to form the α-helical structure, and penetrated the membrane through the pore formation, leading to the cell apoptosis [29].

Moreover, the high-voltage electric field non-thermal plasma produces a large number of charged ions and increases the charges on the surface of membrane cells. The cellular transmembrane potential difference then increases [30]. As mentioned above, the bacteriocin performs with the help of electrostatic interaction, which is a precondition for the entrance of bacteriocin into phospholipid bilayer. The intermolecular force can be enhanced by the improvement of charges on cell membrane surface caused by non-thermal plasma [31]. This explained the results that the synergistic treatment by lactocin C-M2 and NTPS injured *Morganella* sp. wf-1 cells in a larger degree than the C-M2 (Figure 3 and Figure 7). As reported by Yasuda [32], the damage on bacteria was also caused by the reactive groups generated from the NTPS. These oxidative-stress-related substances entered through the surface pores into the cell membrane in the exposure of bacteriocin. Thus, the lipid bonds on the glycerin-linked side chains of the cell membrane were more easily attacked by the hydroxyl radicals and then ruptured. The composition of fatty acids and the SFA/UFA ratio varied for *Morganella* sp. wf-1 cells treated by the combination of C-M2 and NTPS (Table 1 and Figure 6).

To further interpret the effects based on the perforation theory, the leakage of K^+^, proteins, and DNA/RNA was investigated. The bacteria cell membrane is a protective barrier for the permeation of small ions, such as Na^+^, K^+^, H^+^, etc., of great significance for improving the membrane function and maintaining the enzymatic activity [33]. However, as the barrier is destroyed, the leakage of inter-cell molecular increased [22]. In our study, the bacteriocin-generated pores on cell membrane surface opened the channel for inter-cell substances leaking outsides. The permeability as well as UV-absorbing materials was aggrandized in cells treated by C-M2 and/or NTPS (Figure 4 and Figure 5). As previously reported, the other bacteriocins such as bifidin-A, PlnEF, and pentose 31-1 also acted against putrefactive bacteria by increasing the leakage rate of Na^+^, K^+^, ATP, AKP, and proteins [4,10].

The NTPS not only assists for the lactocin to sterilize bacteria cells by accelerating the osmotic pressure, but generates the ROS such as hydroxyl radical and singlet oxygen to destroy the DNA, protease and lipidosome in cells. The oxidative adducts, produced upon the attack of NTPS on the 8^th^ carbon atom of the guanine base in DNA, leads to the inactivation of bacteria [34]. This could explain the result in Figure 8, that the greatest DNA damage on *Morganella* sp. wf-1 occurred in group C-M2 + NTPS, while the damage in group C-M2 only was not significant. On the other hand, the rising amount of ROS affects the cell metabolism and the activity of ATP synthase, consequently blocking the electron transport chain traverse cell membrane. The electrons then packed together in cells and react with oxygen to create radicals [35]. Therefore, the NTPS treatment in the present study could successively injure the DNA and other genetic materials in *Morganella* sp. wf-1 cells by a two-step mechanism.

## 4. Materials and Methods

### 4.1. Chemicals

The plate count agar (PCA) culture medium was purchased from Haibo Biotechnology Ltd. (Qingdao, China), the mannitol salt agar (MSA) and potato dextrose agar (PDA) culture media were from Luqiao Technology Ltd. (Beijing, China). The LIVE/DEAD^®^ Bac Light TM Bacterial Viability Kit, E.Z.N.A.^®^ Bacterial DNA Kit and EpiQuik 8-OHdG DNA Damage Quantification Direct Kit were supported by Thermo Fisher Scientific Inc. (Shanghai, China), Omega Bio-tek Inc. (Beijing, China) and Epigentek Group Inc. (New York, NY, USA) respectively. The propidium iodide (PI) and SYTO 9 stains and the fluorescein isothiocyanate (FITC) were produced by Biotime Biotechnology Ltd. (Shanghai, China). The ethylene diamine tetraacetic acid (EDTA), hydrochloric acid, sodium chloride, and some other chemicals were of analytical grade and were purchased from Sinopharm Chemical Reagent Co., Ltd. (Beijing, China).

### 4.2. Bacterial Strains, Culture Condition, and Preparation

*Morganella* sp. wf-1 was isolated from white fish and conserved in the Food Bioengineering Laboratory, Institute of Agro-product Processing, Jiangsu Academy of Agricultural Sciences, Nanjing, China. *Morganella* sp. wf-1 was first activated in broth culture at 37 °C for 15 h. Cells were harvested by centrifugation at 6000 rpm at 4 °C for 15 min, washed twice, and then res-suspended in sterilized 0.8% (*w*/*v*) sodium chloride saline solution to a final concentration of 10^7^–10^8^ CFU/mL for future use (about 7.67 log_10_ CFU/mL).

### 4.3. Different Treatments by Lactocin C-M2 and NTPS

Different concentrations (0, 0.1, 0.2, 0.3, 0.4, 0.5, 0.6 mg/mL) of Lactocin C-M2 were used against the putrefactive bacteria *Morganella* sp. wf-1 prior to the NTPS at 65 kV for 90 s. The plasma system used is this research was based on previous work [36,37]. A detailed characterization of the devices is given in Supplementary Materials (Figures S1 and S2). Moreover, the NTPS at 65 kV for different durations (0, 30, 60, 90, 120, 150, 180 s) were also set as the conditions, following the addition of 0.3 mg/mL Lactocin C-M2, for the bacteriostasis. The treated bacteria suspension was allowed to stand at 4 °C for 6 h prior to the viable bacterial counting in order to ascertain the sufficient action of ionized gas on the bacteria cells.

A control group without C-M2 and NTPS treatments, used the saline to replace the C-M2 solution. The C-M2 group and NTPS group were set under the treatments, respectively by only C-M2 of 0.3 mg/mL, and by only NTPS at 65 kV for 120 s. The bacteria synergistically treated with C-M2 and NTPS were used as the C-M2 + NTPS group. All the treated bacteria suspensions were allowed to stand at 4 °C for 6 h in prior to further experiments.

### 4.4. Viable Bacterial Counting

After the treatments by C-M2 and NTPS as above, the bacteria suspension was centrifuged at 6000 rpm for 10 min. The cells sediment was collected and suspended in 0.8% (*w*/*w*) physiological saline for a series of ten-fold dilutions. 0.1 mL of cell suspension for each of the three optimal dilutions was then coated on the VRBDA agar medium at 37 °C for 48 h [38]. The confirmed colonies were counted, and the bacterial counts were expressed as log_10_ CFU/mL.

### 4.5. Transmission Electron Microscopy (TEM)

TEM was analyzed using the method by Sharma [39] with some slight modifications. The bacteria cells were harvested under the condition of centrifugation at 6000 rpm for 10 min and processed for fixation. After being washed and dehydrated, the pellets were infiltrated and embedded. The thick sections (1 mm) cut with an ultramicrotome were then mounted onto glass slides and observed under a light microscope for the area and quality of the tissue. For the TEM examination, thin sections (70–80 nm) were cut and stained with the alcoholic uranyl acetate and alkaline lead citrate, followed by gentle washing and observation under a JEM-1200EX transmission electron microscope (Japan Electronics Co., Ltd., Japan) at an operating voltage 120 kV.

### 4.6. Cell Membrane Permeability

To determine the impact of bacteriocin or/and NTPS treatment(s) on cell membrane permeability, 1 mL of *O*-Nitrophenyl-β-d-Galactopyranoside (ONPG, 10 g/L) was added to bacteria suspension. The mixture was homogenized and incubated at 37 °C in a water bath. The completely reacted mixture was then centrifuged at 13,000 rpm for 5 min, and the absorbance of the supernatant was determined at a wavelength of 405 nm [4]. The cell membrane permeability was expressed as the difference of optical density (OD) values between the control and treated groups.

### 4.7. Release of Cell Nucleic Acid, Protein, and K^+^

Bacteria suspension were filtered using a 0.22-μm nitrocellulose membrane. The absorbance of the cell-free filtration was recorded at the wavelength of 260 and 280 nm with a UV-visible spectrophotometer (Genesys 20, Thermo Electron, Waltham, MA, USA) to indicate the amounts of extracellular nucleic acid and protein [4]. The concentration of K^+^ in the supernatant was determined by an atomic absorption spectrometer (AAnalytst 800, Perkin Elmer, Waltham, MA, USA).

### 4.8. Fatty Acid Composition in Cell Membranes

Briefly, 40 mg of the lyophilized bacteria cells was mixed with 1 mL of sodium hydroxide-methanol solution (NaOH: CH_3_OH: H_2_O_2_ = 3:10:10, *w*/*v*/*v*). The mixture was placed in a boiling water bath for 30 min, vortexing for 10 s every five minutes. After the reaction, 2 mL of hydrochloric acid-methanol solution (6 M HCl: CH_3_OH = 13:11, *v*/*v*) was added to the mixture, followed by the incubation at a water bath at 80 °C for 10 min. Then, 1.25 mL of hexane-methyl tertiary butyl ether solution (1:1, *v*/*v*) was added for a 10 min reaction. The lipid phase was then harvested to mix with 3 mL of 1.2% sodium hydroxide (*w*/*v*) for 5 min, prior to the fatty acid analysis using a gas chromatogram (GC, Agilent 7890A, Santa Clara, CA, USA), which was coupled with a flame ionization detector (FID) [40]. The ratios of SFA to UFA were calculated.

### 4.9. Confocal Laser Scanning Microscopy (CLSM)

The bacteria cell suspension was treated according to the instructions of LIVE/DEAD^®^ BacLight Bacterial Vibility Kit (BestBio, Shanghai, China). The collected cells were stained with propidium iodide (PI) and SYTO 9. After being labeled by the fluorescein isothiocyanate (FITC), cells were imaged using a confocal laser scanning microscope (LSM 710, Carl Zeiss, Jena, Germany) [41]. The viable bacteria were stained green and the dead were as red.

### 4.10. DNA Damage in Cells

Briefly, 700 μL of SDS-Tris mixture (10% SDS: 1.5 M Tris = 4:1, *v*/*v*) was added to the cell pellet, followed by the homogenization and the centrifugation at 12000 rpm at 4 °C for 10 min. Then, the supernatant was collected and mixed with the equal-volume phenol to react for 3 min. Two-fold volume of ethanol was added and incubated for 1 h in an ice bath, and then centrifuged to harvest the sediments. After rinsing with 70% ethanol (*v*/*v*), the sediments were centrifuged again to remove the supernatant. After the residual ethanol volatilized, 30 μL of sterilized water was used to resolve the DNA precipitate [22]. The DNA damage was measured according to the instructions of EpiQuik 8-OHdG DNA Damage Quantification Direct Kit (Epigentek, Farmingdale, NY, USA).

### 4.11. Statistical Analysis

All the experiments were performed in triplicates unless otherwise is stated. The results were expressed as the means ± standard deviations (SD) and were analyzed using one-way analysis of variance (ANOVA) with SAS version 8.2 (SAS Institute Inc., Raleigh, NC, USA). The LSD (least-significant difference) tests were conducted to compare the significance among different means at *p* value < 0.05. The figures were obtained with Origin software, version 8.5 (Originlab Corp., Northampton, MA, USA).

## 5. Conclusions

The present study systematically investigated the synergistic effects of bacteriocin from *Lactobacillus panis* C-M2 and dielectric barrier discharged non-thermal plasma (DBD-CP) against the putrefactive bacteria *Morganella* sp. wf-1 isolated from aquatic foods. The results proved that the combination of Lactocin C-M2 and NTPS had a greater influence on the inactivation of *Morganella* sp. wf-1 than the single treatment. As concluded, the morphology of bacteria cells varied among different treated groups, and the C-M2 + NTPS group showed the highest cell membrane permeability. The increased amount of released cell nucleic acid, protein, and K^+^, as well as the decreased SFA/UFA ratio, indicated together that the cell membrane integrity of *Morganella* sp. wf-1 was destroyed maximally in the C-M2 + NTPS group. The bacteriocin C-M2 and NTPS could assist each other to disinfect the putrefactive bacteria in aquatic foods. This study supported the theoretical basis for the application of chemical and non-thermal treatments in the current food industry.

## Figures and Tables

**Table 1:** Fatty acid composition (%) in cell membranes of *Morganella* sp.wf-1 by different treatments.

Saturated Fatty Acids	CK	C-M2	NTPS	C-M2 + NTPS	Unsaturated Fatty Acids	CK	C-M2	NTPS	C-M2 + NTPS
C4:0	5.21	1.64	6.56	5.09	C14:1	6.23	41.72	43.40	46.93
C6:0	0.98	0.39	0.40	0.00	C15:1	0.24	1.64	0.07	1.18
C8:0	0.46	0.00	0.00	0.00	C16:1	0.27	8.63	0.31	26.18
C10:0	0.75	0.39	0.00	0.00	C17:1	0.21	0.61	0.79	0.43
C11:0	0.48	0.00	0.00	0.00	C18:1n9t	4.61	2.94	2.64	2.95
C12:0	0.27	0.00	0.14	0.00	C18:1n9c	45.61	25.98	28.75	8.93
C13:0	0.28	0.74	0.21	0.00	C18:2n6t	0.04	0.88	2.09	0.00
C14:0	0.42	0.99	0.21	0.00	C18:3n6	0.02	0.00	0.02	0.00
C15:0	0.11	0.27	0.18	0.30	C20:1	1.32	0.46	1.09	0.38
C16:0	9.86	3.29	0.20	1.60	C18:3n3 /C21:0	0.52	0.00	0.27	0.00
C17:0	1.29	0.55	0.36	0.00	C20:2	0.39	0.00	0.23	0.00
C18:0	7.03	4.25	4.73	4.15	C20:3n6	0.05	0.00	0.04	0.00
C20:0	3.52	3.68	4.39	1.88	C22:1n9	9.11	0.97	2.43	0.00
C22:0	0.12	0.00	0.04	0.00	C20:3n3 /C20:4n6	0.06	0.00	0.07	0.00
C23:0	0.22	0.00	0.00	0.00	C22:2	0.14	0.00	0.00	0.00
C24:0	0.11	0.00	0.00	0.00	C20:5n3	0.04	0.00	0.07	0.00
					C24:1	0.03	0.00	0.32	0.00

## Supplementary Materials

The following are available online at https://www.mdpi.com/2079-6382/9/9/593/s1, Figure S1: Schematic diagram of DBD system, Figure S2: Optical emission spectra of NTPS ranging from 200 nm to 1000 nm. O (777 nm) and O (844 nm), ∙OH (306–309 nm).
