# Supplementary material for: Synergistic Effects of Bacteriocin from Lactobacillus panis C-M2 Combined with Dielectric Barrier Discharged Non-Thermal Plasma (DBD-NTP) on Morganella sp. in Aquatic Foods
Source: Antibiotics (Basel). 2020 Sep 10;9(9):593. doi: 10.3390/antibiotics9090593 (PMC7557774; doi:10.3390/antibiotics9090593)
Supplement: Supplementary file 1 [file antibiotics-09-00593-s001.pdf]

Supplement Materials

The plasma system used is this research was based on previous work[1-3]. It consisted by Mobile Dielectric Test Sets (Phenix Technologies, MD, USA), two aluminium disk electrodes 150 mm in diameter and dielectric barriers layers (polypropylene sheets). The implement voltage was 65 kV (peak-to-peak) and frequency was 50 Hz. The schematic diagram of DBD system is given in Figure S1. The optical emission spectra of NTPS system is given in Figure S2. This corresponding introduction will be added in the supplement.

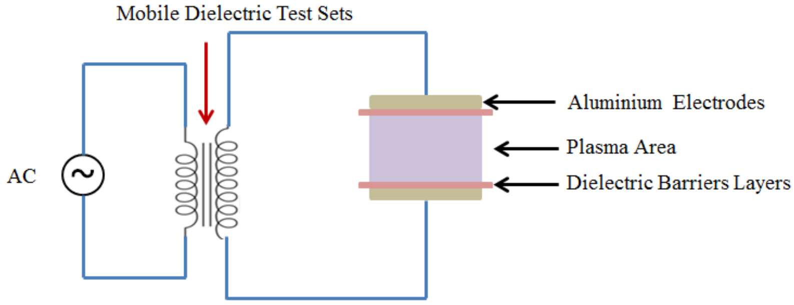

Figure S1. Schematic diagram of DBD system.

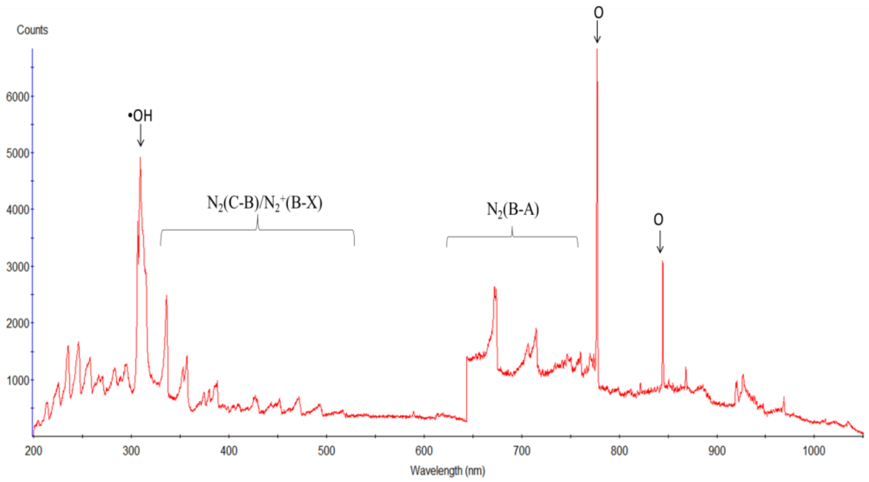

Figure S2. Optical emission spectra of NTPS ranging from 200 nm to 1000 nm. O (777 nm) and O (844 nm), ·OH (306-309 nm).

1. Wang, J.; Zhuang, H.; Hinton, A., Jr.; Zhang, J. Influence of in-package cold plasma treatment on microbiological shelf life and appearance of fresh chicken breast fillets. *Food microbiology.* **2016**, *60*, 142-146, doi:10.1016/j.fm.2016.07.007.
2. Wang, J.; Zhuang, H.; Zhang, J. Inactivation of Spoilage Bacteria in Package by Dielectric Barrier Discharge Atmospheric Cold Plasma—Treatment Time Effects. *Food and Bioprocess Technology.* **2016**, *9*, 1648-1652, doi:10.1007/s11947-016-1746-6.
3. Huang, M.; Wang, J.; Zhuang, H.; Yan, W.; Zhao, J.; Zhang, J. Effect of in-package high voltage dielectric barrier discharge on microbiological, color and oxidation properties of pork in modified atmosphere packaging during storage. *Meat science.* **2019**, *149*, 107-113, doi:10.1016/j.meatsci.2018.11.016.
